# MicroRNAs and Metastasis

**DOI:** 10.3390/cancers12010096

**Published:** 2019-12-30

**Authors:** Carla Solé, Charles H. Lawrie

**Affiliations:** 1 Molecular Oncology Group, Biodonostia Research Institute, 20014 San Sebastián, Spain; carla.sole@biodonostia.org; 2 IKERBASQUE, Basque Foundation for Science, 48013 Bilbao, Spain; 3 Radcliffe Department of Medicine, University of Oxford, Oxford OX3 9DU, UK

**Keywords:** miRNA, metastasis, cancer, liquid biopsies

## Abstract

Metastasis, the development of secondary malignant growths at a distance from the primary site of a cancer, is associated with almost 90% of all cancer deaths, and half of all cancer patients present with some form of metastasis at the time of diagnosis. Consequently, there is a clear clinical need for a better understanding of metastasis. The role of miRNAs in the metastatic process is beginning to be explored. However, much is still to be understood. In this review, we present the accumulating evidence for the importance of miRNAs in metastasis as key regulators of this hallmark of cancer.

## 1. Introduction

Nearly half of all patients with cancer present with some form of metastasis at time of diagnosis [1]. Unfortunately, with very few exceptions, metastatic disease remains essentially incurable and almost 90% of all cancer deaths are associated with metastasis [2,3]. Consequently, there is a clear clinical need for a better understanding of metastasis and the development of novel therapeutics targeting this process. 

In essence, metastasis, the development of secondary malignant growths at a distance from the primary site of a cancer, is a multiphase process that requires tumor cells to detach from the primary tumor mass, enter and travel through the blood or lymph system, to leave circulation, and to form a new tumor in other organs or tissues of the body. The process of metastasis is very inefficient, with the survival rate of circulating tumor cells (CTCs) being as low as 0.2%, and then only those survivors can successfully metastasize target organs only when optimal conditions occur [4]. 

**Figure 1:**
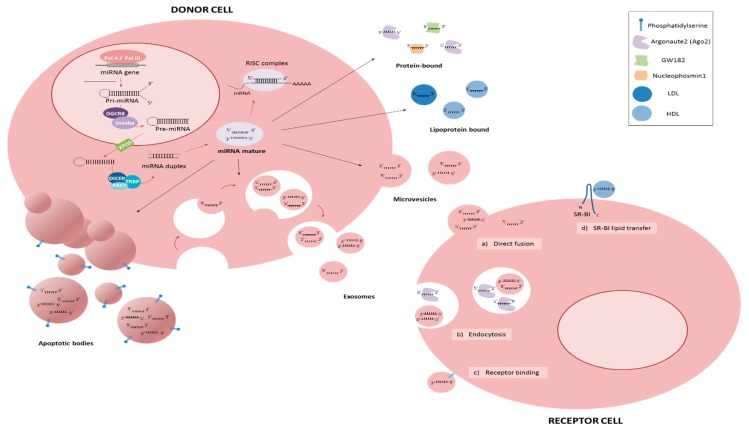
The release of microRNAs (miRNAs) in the extracellular environment. MiRNAs could enter the RNA induced silencing complex (RISC) to regulate mRNA expression and/or translation or can be released outside cell to reach biological fluids. Circulating miRNAs could be found in two major forms, vesicle associated and non-vesicle associated. When miRNAs are released by donor cells through vesicles, these could be microvesicles by outward budding (100–1000 nm), exosomes (50–100 nm) or apoptotic bodies as a result of apoptosis (1–5 μm). On the other hand, cell-free miRNAs could be found bound to proteins, such as Argonaute2 (AGO2), GW182 and Nucleophosmin1 (NPM1), or bound to lipoproteins, high-density lipoproteins (HDLs) or low-density lipoproteins (LDLs) [8]. Circulating miRNAs uptake by receptor cells occurred by membrane direct fusion (**a**), endocytosis (**b**) and receptor binding (**c**), which could trigger a downstream cascade or could produce internalization of the vesicle [9,10]. Moreover, it has been demonstrated that delivery of HDL miRNAs is dependent on scavenger receptor class B type I (SR-BI) [11,12].

MicroRNAs (miRNAs) are small (19–25 nt) non-coding single-stranded RNAs that regulate gene expression through imperfect binding to the 3′ untranslated region (UTR) of target genes [5] (Figure 1). Because a single miRNA can target several hundred genes, and a single target gene often contains multiple miRNA binding sites, it is believed that more than 60% of all human genes are a direct target for miRNA regulation [6]. Consequently, miRNAs have been shown to play key regulatory roles in virtually every aspect of biology including both physiological and pathological processes, most notably in cancer. The importance of miRNAs in controlling cancer development and progression is well established [7]. In this review, we will consider the identity and role of miRNAs in the metastatic process. 

The first reports associating miRNAs with metastasis came in 2007, with the demonstration that *miR-10b* was induced by Twist1 binding and could promote metastasis in breast cancer in vitro and in vivo through targeting of Homeobox D10 (HOXD10) [13]. In the same year, also in breast cancer, *let-7* was identified as a suppressor of metastasis acting to target the GTPase H-RAS and High Mobility Group AT-Hook 2 (*HMGA2*) gene in tumor-initiating cells, resulting in reduced proliferation and mammosphere formation in vitro and decreased metastasis in a NOD/SCID murine model [14]. In subsequent studies, breast cancer has remained the main focus of research investigating miRNAs in metastasis, and many studies have shown that miRNAs can act as both promoters or inhibitors of metastasis in cancer and modulate many steps of the metastatic pathway, including migration, invasion, adhesion, the epithelial–mesenchymal transition (EMT), niche conditioning and proliferation in secondary site (Table 1) [15,16]. 

## 2. Metastasis—Promoting miRNAs

In common with *miR-10b*, many of the metastasis-promoting miRNAs that have been characterized have been identified in breast cancer. For example, *miR-105* has been identified as being up-regulated in tumor cells and exosomes derived from breast cancer cells were demonstrated to breakdown vascular endothelial barriers and induce vascular permeability, thereby promoting metastasis by targeting of *ZO-1*, a component of cell–cell adhesion complexes in endothelial and epithelial cells [17]. Furthermore, these authors used exosomes to reduce tight junction formation in endothelial monolayers and induce vascular permeability and metastasis in vivo. *MiR-181b-3p* was demonstrated to promote EMT in vitro, with its inhibition reducing the expression of mesenchymal markers, migration and invasion in highly metastatic cell lines [18]. *YWHAG* was identified as a direct target of *miR-181b-3p*, which in turn led to protein stabilization of the EMT regulator, *Snail*. The expression of *miR-374a* was found to be up-regulated in patients with distant metastases and poor prognostic outcome. *MiR-374a* A was also demonstrated to promote EMT and metastasis in vitro and in vivo by activation of the Wnt/β-catenin pathway by targeting *WIF1*, *PTEN*, and *WNT5A* which inhibit this cascade [19]. Similarly, *miR-135a* is also highly expressed in metastatic breast cancer and has been demonstrated to promote migration and invasion mediated by targeting *HOXA10* and *APC* [20,21]. The same miRNA is up-regulated in hepatocellular carcinoma (HCC) patients and was found to promote migration and invasion through targeting of *FOXO1* [22]. *MiR-96* is also highly expressed in metastatic breast cancer and was demonstrated to promote cell proliferation, migration and invasion in vitro and enhanced tumor growth in vivo via targeting of *PTPN9* [23]. This miRNA was also observed to be highly expressed in HCC tissue, where it was demonstrated to increase proliferation and migration in vitro through inhibition of *ephrinA5* expression [23]. The hypoxia-induced miRNA, *miR-210*, was found to be up-regulated in breast cancer stem cells (BCSCs) and the expression of this miRNA was shown to promote migration and invasion of these cells mediated by direct targeting of *E-Cadherin* and its transcription repressor, *Snail* [24]. BCSCs were also shown to express high levels of *miR-29a*, which increased levels of migration, invasion and EMT through targeting of the methyltransferase *SUV420H2* [25]. Further, in breast cancer, *miR-130* was found to target *FOSL1* and suppress the inhibition of *ZO-1*, thereby promoting cell migration and invasion [26]. Both *miR-8084* and *miR-1204* were found to enhance migration and invasion through EMT induction via targeting of *ING2* and *VDR* respectively [27,28].

Outside of breast cancer, several other miRNAs have been described to promote EMT. For example, *miR-93* was demonstrated to target *FOXA1* in endometrial carcinoma [29] and *miR-197*, highly up-regulated in metastatic HCC, was found to target *NKD1* and *DKK2*, leading to inhibition of Wnt/β-catenin signaling [30]. In gastric cancer (GC), TGF-β was shown to up-regulate *miR-577* expression, which in turn targets *SDPR*, leading to EMT induction [31]; and *miR-520c*, which was up-regulated in both cell lines and tissues and shown to increase proliferation, migration and invasion of cancer cells through *IRF2* targeting [32]. Similarly, in colorectal cancer (CRC), *miR-1269a* has been found to enhance TGF-β signaling through targeting of *Smad7* and *HOXD10* [33]. 

Esophageal carcinomas have been observed to over-express both *miR-20b* and *miR-9*, which were demonstrated to promote tumorigenic processes including metastasis [34,35]. In particular, the over-expression of *miR-20b* was shown to promote migration and invasion in vitro by targeting and regulation of *PTEN* [34]. In contrast, *miR-9* was shown to stimulate metastasis through promotion of cell migration and induction of the EMT pathway by inhibiting E-Cadherin expression which in turn was demonstrated to induce c-myc and CD44 expression [35]. In non-small-cell lung cancer (NSCLC) *miR-574-5p* was found to promote metastasis in vitro and in vivo through targeting of *TPRU* [36]. In ovarian cancer, miR-194 was demonstrated to increase the growth, migration, and invasion of cells in vitro through targeting of the *PTPN12* gene [37]. 

## 3. Metastasis—Suppressing miRNAs

In addition to miRNAs that promote metastasis, other miRNAs negatively regulate this process and are consequently found to be down-regulated in cancer tissues and/or cell lines. For example, members of the *miR-200* family (i.e., *miR-200a*, *miR-200b*, *miR-200c*, *miR-141* and *miR-429*) as well as *miR-205* have been shown to inhibit the expression of transcription repressors ZEB1 and ZEB2 to enhance E-Cadherin expression, thereby inhibiting EMT in breast cancer [38,39,40]. In a study that looked at miRNA expression in 59 of the NCI60 cell lines that had a E-Cadherin high and vimentin low (EMT inhibitory) phenotype, they observed a strong negative correlation with *miR-200* expression, suggesting that this miRNA is a universal regulator of metastasis in many cancer types including lung, kidney, colon and ovarian cancer [41]. Specifically, *miR-200b* was shown to be down-regulated in triple negative breast cancer (TNBC) as a result of the recruitment of DNMT3A by MYC, which in turn binds to the *miR-200b* promoter region, resulting in promoter methylation and silencing, thereby inhibiting migration, invasion and mammosphere formation in TNBC cells [42]. Part of the *miR-200* family, *miR-141* is also down-regulated in prostate cancer (PC), and its ectopic expression was shown to inhibit invasion and metastasis and to convey a strong epithelial phenotype with a partial mesenchymal phenotype [43]. 

Similar to the *miR-200* family, the *miR-29* family (*miR-29a/b/c*) is another group of negative regulators of metastasis. In head and neck squamous cell carcinoma (HNSCC), these miRNAs were found to inhibit migration and invasion through targeting of the focal adhesion laminin–integrin pathway (LAMC2, ITGA6 and LOXL2) [44,45]. LOXL2 is an enzyme that facilitates metastasis by changing cell morphology and is also regulated by the miR-29 family in clear cell renal cell carcinoma (ccRCC) [46]. LOXL2 is also regulated by *miR-26a/b* and *miR-218* in HNSCC [45], and by *miR-504* in NSCLC [47]. Moreover, one member of the *miR-29* family, *miR-29c*, has been found to be down-regulated in metastatic lung cancer and to reduce adhesion, invasion, migration and metastasis in vitro and in vivo through targeting integrin β1 and metalloproteinase 2 (MMP2), which is involved in extracellular matrix breakdown [48]. 

*miR-203* has also been found to act as a negative regulator of metastasis in several different cancers. In HNSCC, it was shown to inhibit factors involved in cytoskeletal (LASP1), extracellular matrix (SPARC) and metabolic genes (NUAK1) [49]. In breast cancer, *miR-203* regulates EMT via a double-negative feedback loop formed by targeting *TGF-β* and *Slug* [50]. In melanoma, low levels of *miR-203* were associated with poorer overall survival in metastatic patients and its expression in vivo was demonstrated to inhibit metastasis via regulation of Slug [51]. In ovarian cancer cell lines, *miR-203* was also found to be down-regulated and was shown to inhibit the EMT pathway by targeting *BIRC5* and, thereby, attenuating TGFβ activity [52]. In gastric cancer, *miR-203* has been demonstrated to inhibit invasion and EMT through targeting of Annexin A4 [53]. In CRC, *miR-203* expression was linked with clinical stage, lymph node metastasis and poor survival and was demonstrated to regulate cell proliferation, migration and invasion by targeting EIF5A2 expression [54]. 

Similarly, *miR-124* has also been shown to be down-regulated in metastatic CRC when compared with healthy individuals and non-metastatic CRC patients [55]. This miRNA was found to regulate cell proliferation and invasive properties in cell lines through targeting of *ROCK1* expression. *miR-135a* is another down-regulated miRNA that was shown to target ROCK1, thereby inhibiting EMT, invasion and migration of GC cell lines [56]. This miRNA was also observed to be down-regulated in GC patients and its expression was associated with more advance-stage disease and higher rate of lymph node metastases [56]. *ROCK1* expression has also been described to be modulated by *miR-381* in breast cancer, which also regulates other molecules of the Wnt signaling pathway [57]. 

Several miRNAs have been described as metastasis suppressors in breast cancer such as *miR-7*, which was found to regulate FAK and its levels were positively correlated with E-Cadherin expression and negatively correlated with Vimentin and Fibronectin expression [58]. Loss of *miR-31* was associated with invasion and metastasis in breast cancer by regulating genes involved in invasion and metastasis including multiple α subunit partners of β1 and β3 integrins and WAVE3, [59,60]. *MiR-154* is also down-regulated in breast cancer tissues and cell lines where it was shown to inhibit proliferation, migration and invasion by targeting E2F5 [61]. In TNBC, *miR-150* was found to be down-regulated in tumor tissues, and to regulate HMGA2, leading to suppression of migration in vitro [62]. In breast cancer, *miR-124* was observed to be significantly down-regulated in metastatic bone tissues [63]. Recently, nanoparticle delivery of *miR-708*, another inhibitor of metastasis, was shown to reduce lung metastasis in breast cancer in vivo [64]. In another study, *miR-33b* expression was shown to be inversely correlated with the presence of lymph node metastases in breast cancer patients and to inhibit stemness, migration and invasion potential in vitro by targeting HMGA2, SALL4 and Twist1 [65]. Similarly, *miR-34c* was demonstrated to regulate migration and invasion of tumor cells in vitro through targeting of GIT1 [66], a protein whose expression has been linked to the presence of lymph node metastases in breast cancer patients [67]. 

*MiR-34c* and other family members (i.e., *miR-34a/b/c*) have been shown to be induced by activation of p53 and to target Snail, Slug, CD44 and ZEB1 [68]. ZEB1 and ZEB2, and E-Cadherin inhibitors have been demonstrated to be negatively regulated by several miRNAs, including *miR-101* in ovarian carcinoma [69], *miR-139-5p* in glioblastoma multiforme [70], *miR-215* in NSCLC [71] and *miR-132* in CRC and NSCLC [72,73]. 

In addition to direct regulation of metastasis by specific miRNAs, indirect regulation of metastasis can occur by regulation of components of the miRNA biosynthetic machinery. Two of these components, namely Dicer and Drosha, have been shown to be down-regulated in many cancer types [74,75]. It has been found that hypoxia can down-regulate Dicer expression through epigenetic silencing mediated by oxygen-sensitive H3K27me3 demethylases KDM6A and KDM6AB [76]. The authors demonstrated that this global reduction in miRNA expression resulted in down-regulation of *miR-200* which in turn increased levels of ZEB1 regulating metastasis. It has been noted that Dicer is down-regulated in metastatic human tumors deficient in TAp63, which can bind to the Dicer promoter and activates its expression. Deletion of TAp63 in mice reduced Dicer levels in tumors and increased the frequency of metastases [77]. Similarly, another important component of the miRNA biogenesis pathway, AGO2, has been found to be phosphorylated under hypoxic conditions by EGFR in breast cancer cells where it was shown to mediate EGFR-associated tumor cell invasiveness [78]. 

MiRNAs themselves can also directly target biosynthetic components—such is the case for *miR-103*, *miR-107* and *miR-630*. Hypoxia was shown to up-regulate *miR-630* expression, leading to targeting of Dicer [79]. Using an orthotopic murine model of ovarian cancer, the authors demonstrated that delivery of *miR-630* resulted in increased tumor growth and metastasis, along with decreased Dicer expression. In breast cancer, high levels of *miR-103/107* were associated with the presence of metastasis and poor clinical outcome and were demonstrated to directly target Dicer as well as increase the migratory properties of cells in vitro and metastasis in vivo [80]. 

In addition to whole tumors, several studies have looked specifically at the role of miRNAs in cancer stem cells (CSCs) [81,82] which play key roles in metastasis and resistance to therapies [83,84,85,86]. For example, breast CSCs were found to express lower levels of *miR-7* and higher levels of *KLF4*, an essential gene for induced pluripotent stem cells, and the expression of this miRNA was shown to down-regulate metastasis in vitro and in vivo [87,88]. Further identified as being down-regulated in breast CSCs, *miR-4319* was shown to inhibit tumor initiation and metastasis in vivo by targeting E2F2 [89]. In contrast, *miR-31* and *miR-29a* have been found to be up-regulated in breast CSCs, and inhibition of these miRNAs reduced the number and tumor-initiating ability of CSCs along with their metastatic ability 25 [90]. In prostate CSCs, *miR-34a* was found to be down-regulated and restoration of levels of this miRNA inhibited self-renewal capabilities and metastasis through targeting of CD44 [91]. In another study, the ectopic expression of down-regulated *miR-141* in prostate CSCs were demonstrated to inhibit EMT, spheroid formation, invasion and metastatic capabilities via targeting multiple pro-metastatic genes, such as *EZH2*, *CD44* and Rho GTPases [43]. In gastric CSCs, up-regulation of *miR-106b* was shown to enhance self-renewal, invasion and EMT, through activation of the TGF-β/Smad signaling pathway [92].

## 4. Metastasis and Circulating miRNAs

Unlike other RNA types—the vast majority of which are degraded by high levels of RNases found in the blood [93]—miRNAs are stable in the blood and are surprisingly resistant to fragmentation by either chemical or enzymatic agents [94]. Consequently, there has been a great deal of interest in circulating miRNAs in recent years [95]. Although the majority of studies relate to the biomarker potential of circulating miRNAs, they have also been demonstrated to act functionally with the ability to regulate spatially separated cells, a characteristic that lends itself to metastatic regulation [96,97,98]. Indeed, it has been described that cancer cells interact with other cells in the metastatic site to promote their own survival [99,100,101]. MiRNAs can exist in a circulating form either cell-free bound to proteins such as Argonaute2 (Ago2) [102,103], to lipids such as HDLs or LDLs [104], or they can be present inside extracellular vesicles such as exosomes [105,106]. They can act in an autocrine, paracrine and endocrine manner [96]. Several studies have reported higher levels of circulating miRNAs in metastatic patients. For example, *miR-141* levels in serum from prostate cancer patients [94], and levels of *miR-200c* and *miR-141* in breast cancer patients [107].

**Figure 2:**
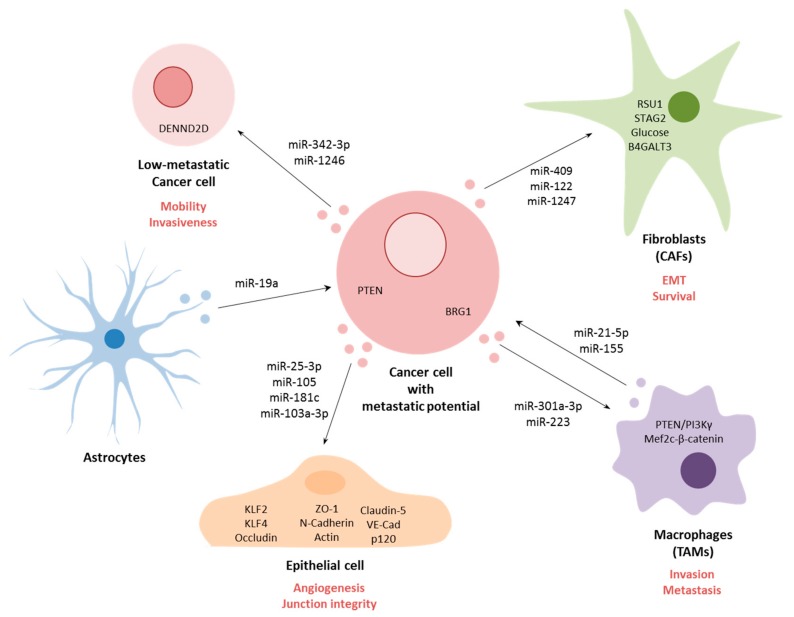
Cancer cell communication through extracellular vesicles (EVs). Cancer cells can communicate with surrounding cells or distant cells via miRNAs contained inside the EVs. Non-tumor cells are usually epithelial cells, macrophages or fibroblasts, although, communication between cancer cells with low-metastatic potential with astrocytes has also been described. Schematic representation of miRNAs involved in each communication and targets described in receptor cells.

Circulating miRNAs have also been found to be present in tumor-secreted extracellular vesicles (EVs), mostly exosomes, which are known to participate in the metastatic process (Figure 2) [108,109,110]. For example, *miR-25-3p*, present in exosomes derived from CRC cells, were demonstrated to enter surrounding epithelial cells and to promote liver and lung metastasis in vivo [111]. The exosome-delivered *miR-25-3p* was shown disrupt the integrity of junctions in epithelial cells and to induce angiogenesis. Furthermore, this effect was mediated through targeting of KLF2, an inhibitor of VEGFR2, thereby decreasing the integrity of the endothelial barrier and targeting related molecule KLF4, leading to the decreased expression of Occludin, Claudin5 and ZO-1—all molecules implicated in maintenance of the cell–cell junction. Similarly, in breast cancer, *miR-105* present in exosomes was demonstrated to target ZO-1, resulting in destruction of vascular structures and enhancing vascular permeability [17]. The authors demonstrated that in vivo exosomal *miR-105* resulted in increased lung and brain metastasis. Furthermore, they observed that serum levels of *miR-105* were higher in patients with distant or lymph node metastasis. In another study, *miR-181c* derived from brain metastasis breast cancer cells could induce abnormal localization of claudin-5, Occludin, ZO-1, N-Cadherin and Actin through transfer of *miR-181c* into blood–brain barrier endothelial cells, resulting in destruction of cell–cell contact [112]. Similarly, levels of exosome-associated miR-181c from breast cancer patient serum were also observed to be significantly higher in patients that suffered brain metastasis. In HCC, when exosomal *miR-103a-3p* was delivered into endothelial cells, the miRNA was shown to abrogate junction integrity and promoted tumor metastasis through targeting of VE-Cad, p120 and ZO-1 [113]. Again, levels of *miR-103a-3p* in serum from HCC patients were associated with higher metastasis potential, higher TNM and higher recurrent risk. 

MiRNAs contained in exosomes have also been shown to influence non-tumor cells in the tumor microenvironment such as tumor-associated macrophages (TAMs) that promote invasion and metastasis in cancer. For example, pancreatic cancer cells under hypoxic conditions were shown to release exosomes that contained *miR-301a-3p*, which was demonstrated to induce TAM polarization resulting in increased pancreatic cell migration and EMT in vitro and lung metastasis in vivo [114]. This polarization was induced by activation of the PTEN/PI3Kγ signaling pathway. In contrast, TAMs themselves have also been shown to secrete exosomes containing functional miRNAs that can promote metastasis. For example, exosomal *miR-223* derived from TAMs of breast cancer patients were demonstrated to promote tumor cell invasion through targeting of the Mef2c-β-catenin pathway [115,116]. In colon cancer, activated TAMs were shown to release exosomes containing *miR-21-5p* and *miR-155-5p*, which were demonstrated to regulate migration and invasion of colorectal cancer cells through targeting of BRG1 [116]. In addition to TAMs, cancer-associated fibroblasts (CAFs), which initiate remodeling of the extracellular matrix, thereby facilitating metastasis, can also release and respond to miRNA-containing exosomes [117]. This is the case for example in prostate cancer, where EV-associated *miR-409* was demonstrated to promote EMT both in vitro and in vivo through down-regulation of RSU1 and STAG2 [118]. In breast cancer, tumor cells were demonstrated to secrete exosomes containing *miR-122* that could induce glucose reallocation in pre-metastatic sites in fibroblast and astrocyte populations, thereby making sites more conducive to metastasizing cancer cells [119]. In liver cancer, tumor-derived exosomal *miR-1247-3p* was shown to promote the activation of fibroblasts to form CAFs through the down-regulation of B4GALT3, leading to activation of the β1-integrin-NF-κB signaling pathway, thereby promoting stemness, EMT, spheroid formation, mobility and chemoresistance in vitro and increasing lung metastasis in vivo [120]. Moreover, higher levels of *miR-1247-3p* were detected in serum from liver cancer patients with lung metastases [120].

Other tumor microenvironment cells have also been shown to be able to communicate with tumor cells as a result of exosome-associated miRNAs. For example, astrocytes in breast cancer patients were found to release exosomes containing *miR-19a* which was demonstrated to regulate PTEN in tumor cells and to promote brain metastasis after tumor extravasation [121]. In oral cancer, highly metastatic tumor cells were demonstrated to release exosomes containing *miR-342-3p* and *miR-1246*, which could be taken up by less metastatic tumor cells, resulting in an increase in their mobility and invasiveness through regulation of DENND2D [122].

## 5. The Metastatic Targetome

As shown above, many miRNAs have now been identified that are associated with the regulation of cancer metastasis. However, the functional significance of such deregulation is poorly understood, as the target genes (the targetome) of miRNAs are notoriously difficult to predict computationally, and moreover differ according to the cellular context [123,124]. An alternative approach is to directly sample the targetome in situ using cross-linking immunoprecipitation (CLIP) techniques coupled with high-throughput sequencing. This technology has evolved through the development of several variations, with arguably the most promising being Photoactivatable-Ribonucleoside-Enhanced CLIP (PAR-CLIP), which has a far better signal-to-noise ratio than other CLIP-based technologies [125]. For example, *miR-200*, a major regulator of cellular migration and invasion, was demonstrated to target *WIPF1*, *CFL2* and *MPRIP* by HITS-CLIP in breast cancer—all genes which promote invadopodia and invasion of cells [126]. In addition, PAR-CLIP was used with prostate cancer cells to show that *miR-148a* reduced migration and invasion by direct interaction with *CENPF* 3′UTR [127]. Similarly, *miR-141* was shown to mediate cell invasion by directly targeting *RAC1*, *CDC42*, and i [43]. Targets of *miR-346* were also identified by CLIP including the oncogene *YWHAZ* that modulates cell invasion and levels were correlated with Gleason grade, biochemical recurrence, non-organ-confined disease and lymph node metastases in patients [128].

## 6. Concluding Remarks and Future Perspectives

As is clear from the evidence presented above (Table 1), many miRNAs play crucial roles in cancer metastasis and, as a result, the therapeutic targeting of these miRNAs has generated a lot of interest in recent years [129,130,131,132,133,134,135]. In general terms, there are two strategies to modulate miRNA expression—either the restoration of down-regulated tumor suppressor miRNAs, or the inhibition of pro-metastatic miRNAs. The former group, requiring the expression of a specific miRNA using techniques such as direct delivery, viral or other vector formats, is much less challenging from a practical point of view than the inhibition of a specific miRNA whose over-expression could be very localized to a few cells or may require complete inhibition to be effective. The latter approach is generally achieved using some type of specific inhibitor such as antagomiRs or miRNA sponges [136,137]. All of these approaches, whether expression or inhibition, face common challenges including poor delivery, low cellular efficiency, endosomal escape and off-target effects, amongst others. Perhaps most attention has been focused on improving delivery systems for miRNAs and/or miRNA inhibitors. Two general approaches have been taken to deliver miRNAs in vivo; viral vectors and non-viral delivery systems. Viral vectors such as lentivirus, adenovirus or adeno-associated viruses [138,139] have been demonstrated to be able to deliver miRNAs with high efficiency in vivo. However, these vectors can trigger an immune response in patients [140]. Consequently, many studies have chosen to use non-viral vectors, in particular nanoparticles such as lipid and polymeric nanoparticles that can protect miRNA from degradation in vivo and, thereby, increase their half-life in circulation but do, however, have much lower transfection efficiencies than viral vectors [141,142,143,144]. For example, lipid-derived nanoparticles carrying *miR-34a* were demonstrated to reduce metastasis and increase survival in an orthotopic model of prostate cancer [91]. Lipid nanoparticles were also used for the systemic delivery of *miR-200* to reduce angiogenesis and metastasis in murine models of ovarian, lung, renal and breast cancer, through regulation of Interleukin 8 and CXCL1 [145]. In NSCLC, cationic liposomes were used to deliver *miR-143* in mice and were demonstrated to inhibit metastasis and prolong survival [146]. CRISPR/Cas9 technology has been used as an alternative to inhibitor sequences in several cancer types [147,148,149]. For example, lentivirus-mediated disruption of *miR-21* by CRISPR-Cas9 technology was shown to inhibit EMT in ovarian cancer [150], and, in glioblastoma, lentivirus-mediated *miR-10b* CRISPR/Cas9 inhibition was found to be lethal for GMB cells and GBM-initiating stem cells both in vitro and in orthotopic mice [151]. In addition to addressing the problem of general delivery, several approaches have been made to improve specific delivery by targeting technologies. For example, *let-7g* was conjugated with an aptamer that binds and antagonizes the oncogenic receptor tyrosine kinase Axl (GL21.T) in lung cell line (A549—Axl+) and breast cancer cell line (MCF7—Axl−) [152]. These constructs were shown to retain cell and tissue specificity in vivo and produce a reduction of tumor volume. Specific cell-targeted aptamers have also been used including delivery of anti-*miR-155* using poly lactic-co-glycolic acid (PLGA) nanoparticles and a peptide with a low pH-induced transmembrane structure (pHLIP) that facilitated the delivery of the inhibitor across the plasma membrane under acidic conditions, such as those found within tumors [153]. This strategy was used in a mouse lymphoma model that led to a reduction of tumor growth without any discernable toxicity. Disulfide-cross-linked polyethylenimine (PEI-SS) was employed along with conjugated folic acid to target in breast cancer [143]. EVs themselves have been used as a vehicle delivery system for metastatic miRNAs. For example, EVs produced by B-cells that contained a *miR-335* synthetic mimic were demonstrated to inhibit *SOX4* expression and to reduce tumor growth in vivo in a breast cancer model [154]. 

In addition to direct modulation of metastasis-associated miRNAs, these delivery systems have also been used to modulate treatment response in a metastatic context. For example, liposomal nanoparticle delivery of anti-*miR-155* was used to reverse cisplatin chemoresistance in a murine lung cancer model of metastasis resulting in reduced proliferation and angiogenesis [155]. In glioblastoma, antagomirs directed against *miR-21* and *miR-10b* were incorporated within nanoparticles (cRGD-tagged PEG-PGLA) and shown to have high levels of uptake by cells and to increase chemosensitivity to Temozolomide in vivo [156]. The same antagomirs were also used for TNBC [157]. Liposomal nanoparticles loaded with *miR-200c* were demonstrated to sensitize metastatic lung cancer cells to radiotherapy in vivo [158]. In addition, multiple studies have been used combining miRNA modulators (mimics or anti-miRNAs) along with chemotherapy—most commonly, doxorubicin with miRNAs such as *let-7a* and *miR-21* in breast cancer [159,160], *miR-31* in cervical cancer [161] and *miR-34a* in prostate cancer [162]. Co-polymer nano-assemblies (PEG5K-VE4-DET20) were co-loaded with *let-7b* mimic and paclitaxel in NSCLC and demonstrated to potentiate the cytotoxicity of paclitaxel and induced apoptosis and inhibition of invasiveness in vivo [163]. Researchers developed a polymeric dual delivery nanoscale device (DDND) to delivery *miR-345* mimic and gemcitabine for metastatic pancreatic cancer [164]. This system was used to reduce tumor growth and decrease metastasis in a murine xenograft model. Gemcitabine was co-delivered with *miR-203a* using an EGFR-targeted cationic polymeric misted micelle system and shown to reduce tumor growth, increase apoptosis and inhibit EMT in an orthotopic pancreatic tumor model [165]. In breast cancer, *miR-34a* and doxorubicin were co-delivered using multi-functional nano-micellar carriers, resulting in reduced tumor formation and metastasis in vivo [166]. 

In addition to these pre-clinical studies, there are a number of clinical trials targeting metastasis-associated miRNAs. For example, *miR-34* mimics encapsulated in liposomal carriers have been intravenously administered to patients with metastatic primary liver cancer, small-cell lung cancer (SCLC), lymphoma, melanoma, multiple myeloma, renal cell carcinoma and NSCLC during a phase 1 trial (MRX34, miRNA Therapeutics Inc.) [167]. However, this trial was terminated before completion in 2016 after serious adverse immune-related effects were developed by some patients (ClinicalTrials.gov identifiers: NCT01829971, NCT02862145). MiRNA Therapeutics have several other miRNA-based clinical trials underway including a phase I trial to deliver a *miR-155* antagomir (MRG-106 or Cobomarsen™), which is currently recruiting patients with lymphoma or leukemia (NCT02580552), and a phase II trial, which is currently recruiting cutaneous T-cell lymphoma patients to compare with Vorinostat treatment (NCT03713320) and a separate follow-up trial (NCT03837457). The MesomiR-1 phase I clinical trial used TargomiR delivery vehicles (bacterially derived minicells containing a targeting antibody and miRNA mimic) to deliver *miR-16* to 26 metastatic pleural mesothelioma patients using an anti-EGFR targeting antibody (ClinicalTrials.gov identifier: NCT02369198) [168,169]. The trial closed in 2017, with a reported objective response of 5% with a duration of 32 weeks. 

In summary, it can be seen from the breadth of evidence presented above that miRNAs (Table 1) represent a key regulatory control of metastasis in multiple cancers and, as a result, are promising targets for novel therapeutic approaches, although it is equally clear that much more research is still needed to translate this knowledge into the clinic. 

## Figures and Tables

**Table 1:** List of miRNAs associated with metastasis.

miRNA	Cancer	Express.	Sample	Target	Ref
*Let-7*	Breast	Low	Cells & Tissue	*RAS & HMGA2*	[14]
*miR-7*	Breast	Low	Cells & Tissue	*FAK*	[58]
*miR-10b*	Breast	High	Cells & Tissue	*HOXD10*	[13]
*miR-26a/b*	HNSCC	Low	Cells & Tissue	*LOXL2*	[45]
*miR-29 family*	HNSCC	Low	Cells	*LAMC2 & ITGA6*	[44]
*miR-29 family*	HNSCC	Low	Cells & Tissue	*LOXL2*	[45]
*miR-29 family*	ccRCC	Low	Cells & Tissue	*LOXL2*	[46]
*miR-29a*	Breast	High	Cells & Tissue	*SUV420H2*	[25]
*miR-29c*	Lung	Low	Cells	*Integrin β1 & MMP2*	[48]
*miR-31*	Breast	Low	Cells	*Integrin α subunits*	[60]
*miR-31*	Breast	Low	Cells & Tissue	*WAVE3*	[59]
*miR-33b*	Breast	Low	Cells & Tissue	*HMGA2, SALL4 & Twist1*	[65]
*miR-34*	CRC	Low	Cells	*ZEB1 & Slug*	[68]
*miR-34a/b/c*	CRC	Low	Cells	*Snail*	[170]
*miR-34a*	Breast	Low	Cells & Tissue	*CXCL10*	[171]
*miR-34c*	Breast	Low	Cells & Tissue	*GIT1*	[66]
*miR-93*	Endometrial	High	Cells & Tissue	*FOXA1*	[29]
*miR-96*	Breast	High	Cells & Tissue	*PTPN9*	[23]
*miR-96*	HCC	High	Cells & Tissue	*ephrinA5*	[172]
*miR-101*	Ovarian	Low	Cells	*ZEB1 & ZEB2*	[69]
*miR-101*	NSCLC	Low	Cells & Tissue	*ZEB1*	[173]
*miR-105*	Breast	High	Cells, Tissue & Serum	*ZO-1*	[17]
*miR-124*	Breast	Low	Cells & Tissue	*ZEB2*	[174]
*miR-124*	Breast	Low	Cells & Tissue	*IL-11*	[63]
*miR-124*	CRC	Low	Cells & Tissue	*ROCK1*	[55]
*miR-128-3p*	ESCC	Low	Cells & Tissue	*ZEB1*	[175]
*miR-130a*	Breast	Low	Cells	*FOSL1*	[26]
*miR-132*	NSCLC	Low	Cells & Tissue	*ZEB2*	[73]
*miR-132*	CRC	Low	Cells & Tissue	*ZEB2*	[72]
*miR-135a*	Breast	High	Cells & Tissue	*HOXA10*	[20]
*miR-135a*	HCC	High	Cells & Tissue	*FOXO1*	[22]
*miR-135a*	Gastric	Low	Cells & Tissue	*ROCK1*	[56]
*miR-135b*	Breast	High	Cells & Tissue	*APC*	[21]
*miR-138*	Breast	Low	Cells & Tissue	*ROCK1*	[57]
*miR-139-5p*	Glioblastoma	Low	Cells & Tissue	*ZEB1 & ZEB2*	[70]
*miR-141*	Breast	Low	Cells & Tissue	*HIPK1*	[176]
*miR-141*	Prostate	Low	Cells & Tissue	*Rho GTases, CD44 & EZH2*	[43]
*miR-150*	Breast	Low	Cells & Tissue	*HMGA2*	[62]
*miR-154*	Breast	Low	Cells & Tissue	*E2F5*	[61]
*miR-181b*	Breast	High	Cells & Tissue	*YWHAG*	[18]
*miR-182*	HCC	High	Cells & Tissue	*ephrinA5*	[172]
*miR-186-5p*	CRC	Low	Cells	*ZEB1*	[177]
*miR-190*	Breast	Low	Cells	*STC2*	[178]
*miR-190*	Breast	Low	Cells & Tissue	*SMAD2*	[179]
*miR-194*	Ovarian	High	Cells & Tissue	*PTPN12*	[37]
*miR-197*	HCC	High	Cells & Tissue	*Axin-2, NKD1 & DKK2*	[30]
*miR-200*	Breast	Low	Cells & Tissue	*ZEB1 & ZEB2*	[39]
*miR-200a/b*	Breast	Low	Cells	*ZEB1 & ZEB2*	[38]
*miR-200c*	Breast	Low	Cells	*CRKL*	[180]
*miR-200c*	Breast	Low	Cells	*ZEB1*	[181]
*miR-200c*	Breast	Low	Cells & Tissue	*HIPK1*	[176]
*miR-203*	HNSCC	Low	Cells & Tissue	*LASP1, SPARC & NUAK*	[49]
*miR-203*	Breast	Low	Cells	*Slug*	[50]
*miR-203*	CRC	Low	Cells & Tissue	*EIF5A2*	[54]
*miR-203*	Melanoma	Low	Cells & Tissue	*Slug*	[51]
*miR-203*	Ovarian	Low	Cells & Tissue	*BIRC5*	[52]
*miR-203*	Gastric	Low	Cells & Tissue	*Annexin A4*	[53]
*miR-205*	Breast	Low	Cells	*ZEB1*	[182]
*miR-205*	Breast	Low	Cells & Tissue	*ZEB1 & ZEB2*	[39]
*miR-210*	Breast	High	Cells & Tissue	*E-Cadherin & Snail*	[24]
*miR-215*	NSCLC	Low	Cells & Tissue	*ZEB2*	[71]
*miR-218*	HNSCC	Low	Cells & Tissue	*LOXL2*	[45]
*miR-374a*	Breast	High	Cells & Tissue	*WIF1, PTEN & WNT5A*	[19]
*miR-409-3p*	Osteosarcoma	Low	Cells & Tissue	*ZEB1*	[183]
*miR-429*	Breast	Low	Cells	*ZEB1 & ZEB2*	[38]
*miR-504*	NSCLC	Low	Cells & Tissue	*LOXL2*	[47]
*miR-508-3p*	Breast	Low	Cells & Tissue	*ZEB1*	[184]
*miR-520c*	Gastic	High	Cells & Tissue	*IRF2*	[32]
*miR-574-5p*	NSCLC	High	Cells, Tissue & Serum	*PTPRU*	[36]
*miR-577*	Gastric	High	Cells & Tissue	*SDPR*	[31]
*miR-641*	Cervical	Low	Cells & Tissue	*ZEB1*	[185]
*miR-708-3p*	Breast	Low	Cells & Tissue	*ZEB1, CDH2 & Vimentin*	[186]
*miR-1204*	Breast	High	Cells & Tissue	*VDR*	[28]
*miR-1269a*	Colorectal	High	Cells & Tissue	*Smad7 & HOXD10*	[33]
*miR-8084*	Breast	High	Cells, Tissue & Serum	*ING2*	[27]

HNSCC; head and neck squamous cell carcinoma; ccRCC, clear cell renal cell carcinoma; CRC, colorectal cancer; HCC, hepatocellular carcinoma; NSCLC, non-small-cell lung carcinoma; ESCC, esophageal cancer.

## References

[1] Winter M., Gibson R., Ruszkiewicz A., Thompson S.K., Thierry B. (2015). Beyond conventional pathology: Towards preoperative and intraoperative lymph node staging. Int. J. Cancer.

[2] Mehlen P., Puisieux A. (2006). Metastasis: A question of life or death. Nat. Rev. Cancer.

[3] Monteiro J., Fodde R. (2010). Cancer stemness and metastasis: Therapeutic consequences and perspectives. Eur. J. Cancer.

[4] Chambers A.F., Groom A.C., MacDonald I.C. (2002). Dissemination and growth of cancer cells in metastatic sites. Nat. Rev. Cancer.

[5] Bartel D.P. (2009). MicroRNAs: Target recognition and regulatory functions. Cell.

[6] Friedman R.C., Farh K.K., Burge C.B., Bartel D.P. (2009). Most mammalian mRNAs are conserved targets of microRNAs. Genome Res..

[7] Lawrie C.H. (2014). MicroRNAs in Medicine.

[8] Cui M., Wang H., Yao X., Zhang D., Xie Y., Cui R., Zhang X. (2019). Circulating MicroRNAs in Cancer: Potential and Challenge. Front. Genet..

[9] Mulcahy L.A., Pink R.C., Carter D.R. (2014). Routes and mechanisms of extracellular vesicle uptake. J. Extracell. Vesicles.

[10] Gangoda L., Boukouris S., Liem M., Kalra H., Mathivanan S. (2015). Extracellular vesicles including exosomes are mediators of signal transduction: Are they protective or pathogenic?. Proteomics.

[11] Shen W.J., Azhar S., Kraemer F.B. (2018). SR-B1: A Unique Multifunctional Receptor for Cholesterol Influx and Efflux. Annu. Rev. Physiol..

[12] Vickers K.C., Palmisano B.T., Shoucri B.M., Shamburek R.D., Remaley A.T. (2011). MicroRNAs are transported in plasma and delivered to recipient cells by high-density lipoproteins. Nat. Cell Biol..

[13] Ma L., Teruya-Feldstein J., Weinberg R.A. (2007). Tumour invasion and metastasis initiated by microRNA-10b in breast cancer. Nature.

[14] Yu F., Yao H., Zhu P., Zhang X., Pan Q., Gong C., Huang Y., Hu X., Su F., Lieberman J. (2007). let-7 regulates self renewal and tumorigenicity of breast cancer cells. Cell.

[15] Nicoloso M.S., Spizzo R., Shimizu M., Rossi S., Calin G.A. (2009). MicroRNAs—The micro steering wheel of tumour metastases. Nat. Rev. Cancer.

[16] Ma L., Weinberg R.A. (2008). Micromanagers of malignancy: Role of microRNAs in regulating metastasis. Trends Genet. Genet..

[17] Zhou W., Fong M.Y., Min Y., Somlo G., Liu L., Palomares M.R., Yu Y., Chow A., O’Connor S.T., Chin A.R. (2014). Cancer-secreted miR-105 destroys vascular endothelial barriers to promote metastasis. Cancer Cell.

[18] Yoo J.O., Kwak S.Y., An H.J., Bae I.H., Park M.J., Han Y.H. (2016). miR-181b-3p promotes epithelial-mesenchymal transition in breast cancer cells through Snail stabilization by directly targeting YWHAG. Biochim. Biophys. Acta.

[19] Cai J., Guan H., Fang L., Yang Y., Zhu X., Yuan J., Wu J., Li M. (2013). MicroRNA-374a activates Wnt/beta-catenin signaling to promote breast cancer metastasis. J. Clin. Investig..

[20] Chen Y., Zhang J., Wang H., Zhao J., Xu C., Du Y., Luo X., Zheng F., Liu R., Zhang H. (2012). miRNA-135a promotes breast cancer cell migration and invasion by targeting HOXA10. BMC Cancer.

[21] Lv Z.D., Xin H.N., Yang Z.C., Wang W.J., Dong J.J., Jin L.Y., Li F.N. (2019). miR-135b promotes proliferation and metastasis by targeting APC in triple-negative breast cancer. J. Cell. Physiol..

[22] Zeng Y.B., Liang X.H., Zhang G.X., Jiang N., Zhang T., Huang J.Y., Zhang L., Zeng X.C. (2016). miRNA-135a promotes hepatocellular carcinoma cell migration and invasion by targeting forkhead box O1. Cancer Cell Int..

[23] Hong Y., Liang H., Uzair Ur R., Wang Y., Zhang W., Zhou Y., Chen S., Yu M., Cui S., Liu M. (2016). miR-96 promotes cell proliferation, migration and invasion by targeting PTPN9 in breast cancer. Sci. Rep..

[24] Tang T., Yang Z., Zhu Q., Wu Y., Sun K., Alahdal M., Zhang Y., Xing Y., Shen Y., Xia T. (2018). Up-regulation of miR-210 induced by a hypoxic microenvironment promotes breast cancer stem cells metastasis, proliferation, and self-renewal by targeting E-cadherin. FASEB J..

[25] Wu Y., Shi W., Tang T., Wang Y., Yin X., Chen Y., Zhang Y., Xing Y., Shen Y., Xia T. (2019). miR-29a contributes to breast cancer cells epithelial-mesenchymal transition, migration, and invasion via down-regulating histone H4K20 trimethylation through directly targeting SUV420H2. Cell Death Dis..

[26] Chen X., Zhao M., Huang J., Li Y., Wang S., Harrington C.A., Qian D.Z., Sun X.X., Dai M.S. (2018). microRNA-130a suppresses breast cancer cell migration and invasion by targeting FOSL1 and upregulating ZO-1. J. Cell. Biochem..

[27] Gao Y., Ma H., Gao C., Lv Y., Chen X., Xu R., Sun M., Liu X., Lu X., Pei X. (2018). Tumor-promoting properties of miR-8084 in breast cancer through enhancing proliferation, suppressing apoptosis and inducing epithelial-mesenchymal transition. J. Transl. Med..

[28] Liu X., Bi L., Wang Q., Wen M., Li C., Ren Y., Jiao Q., Mao J.H., Wang C., Wei G. (2018). miR-1204 targets VDR to promotes epithelial-mesenchymal transition and metastasis in breast cancer. Oncogene.

[29] Chen S., Chen X., Sun K.X., Xiu Y.L., Liu B.L., Feng M.X., Sang X.B., Zhao Y. (2016). MicroRNA-93 Promotes Epithelial-Mesenchymal Transition of Endometrial Carcinoma Cells. PLoS ONE.

[30] Hu Z., Wang P., Lin J., Zheng X., Yang F., Zhang G., Chen D., Xie J., Gao Z., Peng L. (2018). MicroRNA-197 Promotes Metastasis of Hepatocellular Carcinoma by Activating Wnt/beta-Catenin Signaling. Cell. Physiol. Biochem. Int. J. Exp. Cell. Physiol. Biochem. Pharm..

[31] Luo Y., Wu J., Wu Q., Li X., Wu J., Zhang J., Rong X., Rao J., Liao Y., Bin J. (2019). miR-577 Regulates TGF-beta Induced Cancer Progression through a SDPR-Modulated Positive-Feedback Loop with ERK-NF-kappaB in Gastric Cancer. Mol. Ther. J. Am. Soc. Gene Ther..

[32] Li Y.R., Wen L.Q., Wang Y., Zhou T.C., Ma N., Hou Z.H., Jiang Z.P. (2016). MicroRNA-520c enhances cell proliferation, migration, and invasion by suppressing IRF2 in gastric cancer. FEBS Openbio.

[33] Bu P., Wang L., Chen K.Y., Rakhilin N., Sun J., Closa A., Tung K.L., King S., Kristine Varanko A., Xu Y. (2015). miR-1269 promotes metastasis and forms a positive feedback loop with TGF-beta. Nat. Commun..

[34] Wang B., Yang J., Xiao B. (2016). MicroRNA-20b (miR-20b) Promotes the Proliferation, Migration, Invasion, and Tumorigenicity in Esophageal Cancer Cells via the Regulation of Phosphatase and Tensin Homologue Expression. PLoS ONE.

[35] Song Y., Li J., Zhu Y., Dai Y., Zeng T., Liu L., Li J., Wang H., Qin Y., Zeng M. (2014). MicroRNA-9 promotes tumor metastasis via repressing E-cadherin in esophageal squamous cell carcinoma. Oncotarget.

[36] Zhou R., Zhou X., Yin Z., Guo J., Hu T., Jiang S., Liu L., Dong X., Zhang S., Wu G. (2016). MicroRNA-574-5p promotes metastasis of non-small cell lung cancer by targeting PTPRU. Sci. Rep..

[37] Liang T., Li L., Cheng Y., Ren C., Zhang G. (2016). MicroRNA-194 promotes the growth, migration, and invasion of ovarian carcinoma cells by targeting protein tyrosine phosphatase nonreceptor type 12. Onco Targets Ther..

[38] Bracken C.P., Gregory P.A., Kolesnikoff N., Bert A.G., Wang J., Shannon M.F., Goodall G.J. (2008). A double-negative feedback loop between ZEB1-SIP1 and the microRNA-200 family regulates epithelial-mesenchymal transition. Cancer Res..

[39] Gregory P.A., Bert A.G., Paterson E.L., Barry S.C., Tsykin A., Farshid G., Vadas M.A., Khew-Goodall Y., Goodall G.J. (2008). The miR-200 family and miR-205 regulate epithelial to mesenchymal transition by targeting ZEB1 and SIP1. Nat. Cell Biol..

[40] Korpal M., Lee E.S., Hu G., Kang Y. (2008). The miR-200 family inhibits epithelial-mesenchymal transition and cancer cell migration by direct targeting of E-cadherin transcriptional repressors ZEB1 and ZEB2. J. Biol. Chem..

[41] Park S.M., Gaur A.B., Lengyel E., Peter M.E. (2008). The miR-200 family determines the epithelial phenotype of cancer cells by targeting the E-cadherin repressors ZEB1 and ZEB2. Genes Dev..

[42] Pang Y., Liu J., Li X., Xiao G., Wang H., Yang G., Li Y., Tang S.C., Qin S., Du N. (2018). MYC and DNMT3A-mediated DNA methylation represses microRNA-200b in triple negative breast cancer. J. Cell. Mol. Med..

[43] Liu C., Liu R., Zhang D., Deng Q., Liu B., Chao H.P., Rycaj K., Takata Y., Lin K., Lu Y. (2017). MicroRNA-141 suppresses prostate cancer stem cells and metastasis by targeting a cohort of pro-metastasis genes. Nat. Commun..

[44] Kinoshita T., Nohata N., Hanazawa T., Kikkawa N., Yamamoto N., Yoshino H., Itesako T., Enokida H., Nakagawa M., Okamoto Y. (2013). Tumour-suppressive microRNA-29s inhibit cancer cell migration and invasion by targeting laminin-integrin signalling in head and neck squamous cell carcinoma. Br. J. Cancer.

[45] Fukumoto I., Kikkawa N., Matsushita R., Kato M., Kurozumi A., Nishikawa R., Goto Y., Koshizuka K., Hanazawa T., Enokida H. (2016). Tumor-suppressive microRNAs (miR-26a/b, miR-29a/b/c and miR-218) concertedly suppressed metastasis-promoting LOXL2 in head and neck squamous cell carcinoma. J. Hum. Genet..

[46] Nishikawa R., Chiyomaru T., Enokida H., Inoguchi S., Ishihara T., Matsushita R., Goto Y., Fukumoto I., Nakagawa M., Seki N. (2015). Tumour-suppressive microRNA-29s directly regulate LOXL2 expression and inhibit cancer cell migration and invasion in renal cell carcinoma. Febs Lett..

[47] Ye M.F., Zhang J.G., Guo T.X., Pan X.J. (2018). MiR-504 inhibits cell proliferation and invasion by targeting LOXL2 in non small cell lung cancer. Biomed. Pharm..

[48] Wang H., Zhu Y., Zhao M., Wu C., Zhang P., Tang L., Zhang H., Chen X., Yang Y., Liu G. (2013). miRNA-29c suppresses lung cancer cell adhesion to extracellular matrix and metastasis by targeting integrin beta1 and matrix metalloproteinase2 (MMP2). PLoS ONE.

[49] Benaich N., Woodhouse S., Goldie S.J., Mishra A., Quist S.R., Watt F.M. (2014). Rewiring of an epithelial differentiation factor, miR-203, to inhibit human squamous cell carcinoma metastasis. Cell Rep..

[50] Ding X., Park S.I., McCauley L.K., Wang C.Y. (2013). Signaling between transforming growth factor beta (TGF-beta) and transcription factor SNAI2 represses expression of microRNA miR-203 to promote epithelial-mesenchymal transition and tumor metastasis. J. Biol. Chem..

[51] Lohcharoenkal W., Das Mahapatra K., Pasquali L., Crudden C., Kular L., Akkaya Ulum Y.Z., Zhang L., Xu Landen N., Girnita L., Jagodic M. (2018). Genome-Wide Screen for MicroRNAs Reveals a Role for miR-203 in Melanoma Metastasis. J. Investig. Dermatol..

[52] Wang B., Li X., Zhao G., Yan H., Dong P., Watari H., Sims M., Li W., Pfeffer L.M., Guo Y. (2018). miR-203 inhibits ovarian tumor metastasis by targeting BIRC5 and attenuating the TGFbeta pathway. J. Exp. Clin. Cancer Res..

[53] Li J., Zhang B., Cui J., Liang Z., Liu K. (2019). miR-203 inhibits the invasion and EMT of gastric cancer cells by directly targeting Annexin A4. Oncol. Res..

[54] Deng B., Wang B., Fang J., Zhu X., Cao Z., Lin Q., Zhou L., Sun X. (2016). MiRNA-203 suppresses cell proliferation, migration and invasion in colorectal cancer via targeting of EIF5A2. Sci. Rep..

[55] Xi Z.W., Xin S.Y., Zhou L.Q., Yuan H.X., Wang Q., Chen K.X. (2015). Downregulation of rho-associated protein kinase 1 by miR-124 in colorectal cancer. World J. Gastroenterol..

[56] Shin J.Y., Kim Y.I., Cho S.J., Lee M.K., Kook M.C., Lee J.H., Lee S.S., Ashktorab H., Smoot D.T., Ryu K.W. (2014). MicroRNA 135a suppresses lymph node metastasis through down-regulation of ROCK1 in early gastric cancer. PLoS ONE.

[57] Mohammadi-Yeganeh S., Hosseini V., Paryan M. (2019). Wnt pathway targeting reduces triple-negative breast cancer aggressiveness through miRNA regulation in vitro and in vivo. J. Cell. Physiol..

[58] Kong X., Li G., Yuan Y., He Y., Wu X., Zhang W., Wu Z., Chen T., Wu W., Lobie P.E. (2012). MicroRNA-7 inhibits epithelial-to-mesenchymal transition and metastasis of breast cancer cells via targeting FAK expression. PLoS ONE.

[59] Sossey-Alaoui K., Downs-Kelly E., Das M., Izem L., Tubbs R., Plow E.F. (2011). WAVE3, an actin remodeling protein, is regulated by the metastasis suppressor microRNA, miR-31, during the invasion-metastasis cascade. Int. J. Cancer.

[60] Augoff K., Das M., Bialkowska K., McCue B., Plow E.F., Sossey-Alaoui K. (2011). miR-31 is a broad regulator of beta1-integrin expression and function in cancer cells. Mol. Cancer Res..

[61] Xu H., Fei D., Zong S., Fan Z. (2016). MicroRNA-154 inhibits growth and invasion of breast cancer cells through targeting E2F5. Am. J. Transl. Res..

[62] Tang W., Xu P., Wang H., Niu Z., Zhu D., Lin Q., Tang L., Ren L. (2018). MicroRNA-150 suppresses triple-negative breast cancer metastasis through targeting HMGA2. Onco Targets Ther..

[63] Cai W.L., Huang W.D., Li B., Chen T.R., Li Z.X., Zhao C.L., Li H.Y., Wu Y.M., Yan W.J., Xiao J.R. (2018). microRNA-124 inhibits bone metastasis of breast cancer by repressing Interleukin-11. Mol. Cancer.

[64] Ramchandani D., Lee S.K., Yomtoubian S., Han M.S., Tung C.H., Mittal V. (2019). Nanoparticle Delivery of miR-708 Mimetic Impairs Breast Cancer Metastasis. Mol. Cancer Ther..

[65] Lin Y., Liu A.Y., Fan C., Zheng H., Li Y., Zhang C., Wu S., Yu D., Huang Z., Liu F. (2015). MicroRNA-33b Inhibits Breast Cancer Metastasis by Targeting HMGA2, SALL4 and Twist1. Sci. Rep..

[66] Tao W.Y., Wang C.Y., Sun Y.H., Su Y.H., Pang D., Zhang G.Q. (2016). MicroRNA-34c Suppresses Breast Cancer Migration and Invasion by Targeting GIT1. J. Cancer.

[67] Goicoechea I., Rezola R., Arestin M., Caffarel M., Cortazar A.R., Manterola L., Fernandez-Mercado M., Armesto M., Sole C., Larrea E. (2017). Spatial intratumoural heterogeneity in the expression of GIT1 is associated with poor prognostic outcome in oestrogen receptor positive breast cancer patients with synchronous lymph node metastases. F1000Research.

[68] Siemens H., Jackstadt R., Hunten S., Kaller M., Menssen A., Gotz U., Hermeking H. (2011). miR-34 and SNAIL form a double-negative feedback loop to regulate epithelial-mesenchymal transitions. Cell Cycle.

[69] Guo F., Cogdell D., Hu L., Yang D., Sood A.K., Xue F., Zhang W. (2014). MiR-101 suppresses the epithelial-to-mesenchymal transition by targeting ZEB1 and ZEB2 in ovarian carcinoma. Oncol. Rep..

[70] Yue S., Wang L., Zhang H., Min Y., Lou Y., Sun H., Jiang Y., Zhang W., Liang A., Guo Y. (2015). miR-139-5p suppresses cancer cell migration and invasion through targeting ZEB1 and ZEB2 in GBM. Tumour Biol. J. Int. Soc. Oncodevelopmental Biol. Med..

[71] Hou Y., Zhen J., Xu X., Zhen K., Zhu B., Pan R., Zhao C. (2015). miR-215 functions as a tumor suppressor and directly targets ZEB2 in human non-small cell lung cancer. Oncol. Lett..

[72] Zheng Y.B., Luo H.P., Shi Q., Hao Z.N., Ding Y., Wang Q.S., Li S.B., Xiao G.C., Tong S.L. (2014). miR-132 inhibits colorectal cancer invasion and metastasis via directly targeting ZEB2. World J. Gastroenterol..

[73] You J., Li Y., Fang N., Liu B., Zu L., Chang R., Li X., Zhou Q. (2014). MiR-132 suppresses the migration and invasion of lung cancer cells via targeting the EMT regulator ZEB2. PLoS ONE.

[74] Merritt W.M., Lin Y.G., Han L.Y., Kamat A.A., Spannuth W.A., Schmandt R., Urbauer D., Pennacchio L.A., Cheng J.F., Nick A.M. (2008). Dicer, Drosha, and outcomes in patients with ovarian cancer. N. Engl. J. Med..

[75] Lawrie C.H., Cooper C.D., Ballabio E., Chi J., Tramonti D., Hatton C.S. (2009). Aberrant expression of microRNA biosynthetic pathway components is a common feature of haematological malignancy. Br. J. Haematol..

[76] Van den Beucken T., Koch E., Chu K., Rupaimoole R., Prickaerts P., Adriaens M., Voncken J.W., Harris A.L., Buffa F.M., Haider S. (2014). Hypoxia promotes stem cell phenotypes and poor prognosis through epigenetic regulation of DICER. Nat. Commun..

[77] Su X., Chakravarti D., Cho M.S., Liu L., Gi Y.J., Lin Y.L., Leung M.L., El-Naggar A., Creighton C.J., Suraokar M.B. (2010). TAp63 suppresses metastasis through coordinate regulation of Dicer and miRNAs. Nature.

[78] Shen J., Xia W., Khotskaya Y.B., Huo L., Nakanishi K., Lim S.O., Du Y., Wang Y., Chang W.C., Chen C.H. (2013). EGFR modulates microRNA maturation in response to hypoxia through phosphorylation of AGO2. Nature.

[79] Rupaimoole R., Ivan C., Yang D., Gharpure K.M., Wu S.Y., Pecot C.V., Previs R.A., Nagaraja A.S., Armaiz-Pena G.N., McGuire M. (2016). Hypoxia-upregulated microRNA-630 targets Dicer, leading to increased tumor progression. Oncogene.

[80] Martello G., Rosato A., Ferrari F., Manfrin A., Cordenonsi M., Dupont S., Enzo E., Guzzardo V., Rondina M., Spruce T. (2010). A MicroRNA targeting dicer for metastasis control. Cell.

[81] Garg M. (2015). Emerging role of microRNAs in cancer stem cells: Implications in cancer therapy. World J. Stem Cells.

[82] Asadzadeh Z., Mansoori B., Mohammadi A., Aghajani M., Haji-Asgarzadeh K., Safarzadeh E., Mokhtarzadeh A., Duijf P.H.G., Baradaran B. (2019). microRNAs in cancer stem cells: Biology, pathways, and therapeutic opportunities. J. Cell. Physiol..

[83] Clevers H. (2011). The cancer stem cell: Premises, promises and challenges. Nat. Med..

[84] Yu Z., Pestell T.G., Lisanti M.P., Pestell R.G. (2012). Cancer stem cells. Int. J. Biochem. Cell Biol..

[85] Seton-Rogers S. (2013). Cancer stem cells: Easily moulded. Nat. Rev. Cancer.

[86] Prasetyanti P.R., Medema J.P. (2017). Intra-tumor heterogeneity from a cancer stem cell perspective. Mol. Cancer.

[87] Okuda H., Xing F., Pandey P.R., Sharma S., Watabe M., Pai S.K., Mo Y.Y., Iiizumi-Gairani M., Hirota S., Liu Y. (2013). miR-7 suppresses brain metastasis of breast cancer stem-like cells by modulating KLF4. Cancer Res..

[88] Zhang H., Cai K., Wang J., Wang X., Cheng K., Shi F., Jiang L., Zhang Y., Dou J. (2014). MiR-7, inhibited indirectly by lincRNA HOTAIR, directly inhibits SETDB1 and reverses the EMT of breast cancer stem cells by downregulating the STAT3 pathway. Stem Cells.

[89] Chu J., Li Y., Fan X., Ma J., Li J., Lu G., Zhang Y., Huang Y., Li W., Huang X. (2018). MiR-4319 Suppress the Malignancy of Triple-Negative Breast Cancer by Regulating Self-Renewal and Tumorigenesis of Stem Cells. Cell. Physiol. Biochem. Int. J. Exp. Cell. Physiol. Biochem. Pharmacol..

[90] Lv C., Li F., Li X., Tian Y., Zhang Y., Sheng X., Song Y., Meng Q., Yuan S., Luan L. (2017). MiR-31 promotes mammary stem cell expansion and breast tumorigenesis by suppressing Wnt signaling antagonists. Nat. Commun..

[91] Liu C., Kelnar K., Liu B., Chen X., Calhoun-Davis T., Li H., Patrawala L., Yan H., Jeter C., Honorio S. (2011). The microRNA miR-34a inhibits prostate cancer stem cells and metastasis by directly repressing CD44. Nat. Med..

[92] Yu D., Shin H.S., Lee Y.S., Lee Y.C. (2014). miR-106b modulates cancer stem cell characteristics through TGF-beta/Smad signaling in CD44-positive gastric cancer cells. Lab. Investig. A J. Tech. Methods Pathol..

[93] Duttagupta R., Jiang R., Gollub J., Getts R.C., Jones K.W. (2011). Impact of Cellular miRNAs on Circulating miRNA Biomarker Signatures. PLoS ONE.

[94] Mitchell P.S., Parkin R.K., Kroh E.M., Fritz B.R., Wyman S.K., Pogosova-Agadjanyan E.L., Peterson A., Noteboom J., O’Briant K.C., Allen A. (2008). Circulating microRNAs as stable blood-based markers for cancer detection. Proc. Natl. Acad. Sci. USA.

[95] Fernandez-Mercado M., Manterola L., Larrea E., Goicoechea I., Arestin M., Armesto M., Otaegui D., Lawrie C.H. (2015). The circulating transcriptome as a source of non-invasive cancer biomarkers: Concepts and controversies of non-coding and coding RNA in body fluids. J. Cell. Mol. Med..

[96] Cortez M.A., Bueso-Ramos C., Ferdin J., Lopez-Berestein G., Sood A.K., Calin G.A. (2011). MicroRNAs in body fluids—The mix of hormones and biomarkers. Nat. Rev. Clin. Oncol..

[97] Weber J.A., Baxter D.H., Zhang S., Huang D.Y., Huang K.H., Lee M.J., Galas D.J., Wang K. (2010). The microRNA spectrum in 12 body fluids. Clin. Chem..

[98] Sole C., Arnaiz E., Manterola L., Otaegui D., Lawrie C.H. (2019). The circulating transcriptome as a source of cancer liquid biopsy biomarkers. Semin. Cancer Biol..

[99] Aguirre-Ghiso J.A. (2010). On the theory of tumor self-seeding: Implications for metastasis progression in humans. Breast Cancer Res..

[100] Fidler I.J. (2003). The pathogenesis of cancer metastasis: The ‘seed and soil’ hypothesis revisited. Nat. Rev. Cancer.

[101] Aguirre-Ghiso J.A. (2018). How dormant cancer persists and reawakens. Science.

[102] Arroyo J.D., Chevillet J.R., Kroh E.M., Ruf I.K., Pritchard C.C., Gibson D.F., Mitchell P.S., Bennett C.F., Pogosova-Agadjanyan E.L., Stirewalt D.L. (2011). Argonaute2 complexes carry a population of circulating microRNAs independent of vesicles in human plasma. Proc. Natl. Acad. Sci. USA.

[103] Turchinovich A., Weiz L., Langheinz A., Burwinkel B. (2011). Characterization of extracellular circulating microRNA. Nucleic Acids Res..

[104] Vickers K.C., Remaley A.T. (2012). Lipid-based carriers of microRNAs and intercellular communication. Curr. Opin. Lipidol..

[105] Stoorvogel W. (2012). Functional transfer of microRNA by exosomes. Blood.

[106] Caby M.P., Lankar D., Vincendeau-Scherrer C., Raposo G., Bonnerot C. (2005). Exosomal-like vesicles are present in human blood plasma. Int. Immunol..

[107] Zhang G., Zhang W., Li B., Stringer-Reasor E., Chu C., Sun L., Bae S., Chen D., Wei S., Jiao K. (2017). MicroRNA-200c and microRNA- 141 are regulated by a FOXP3-KAT2B axis and associated with tumor metastasis in breast cancer. Breast Cancer Res..

[108] Tominaga N., Katsuda T., Ochiya T. (2015). Micromanaging of tumor metastasis by extracellular vesicles. Semin. Cell Dev. Biol..

[109] Hoshino A., Costa-Silva B., Shen T.L., Rodrigues G., Hashimoto A., Tesic Mark M., Molina H., Kohsaka S., Di Giannatale A., Ceder S. (2015). Tumour exosome integrins determine organotropic metastasis. Nature.

[110] Peinado H., Lavotshkin S., Lyden D. (2011). The secreted factors responsible for pre-metastatic niche formation: Old sayings and new thoughts. Semin. Cancer Biol..

[111] Zeng Z., Li Y., Pan Y., Lan X., Song F., Sun J., Zhou K., Liu X., Ren X., Wang F. (2018). Cancer-derived exosomal miR-25-3p promotes pre-metastatic niche formation by inducing vascular permeability and angiogenesis. Nat. Commun..

[112] Tominaga N., Kosaka N., Ono M., Katsuda T., Yoshioka Y., Tamura K., Lotvall J., Nakagama H., Ochiya T. (2015). Brain metastatic cancer cells release microRNA-181c-containing extracellular vesicles capable of destructing blood-brain barrier. Nat. Commun..

[113] Fang J.H., Zhang Z.J., Shang L.R., Luo Y.W., Lin Y.F., Yuan Y., Zhuang S.M. (2018). Hepatoma cell-secreted exosomal microRNA-103 increases vascular permeability and promotes metastasis by targeting junction proteins. Hepatology.

[114] Wang X., Luo G., Zhang K., Cao J., Huang C., Jiang T., Liu B., Su L., Qiu Z. (2018). Hypoxic Tumor-Derived Exosomal miR-301a Mediates M2 Macrophage Polarization via PTEN/PI3Kgamma to Promote Pancreatic Cancer Metastasis. Cancer Res..

[115] Yang M., Chen J., Su F., Yu B., Su F., Lin L., Liu Y., Huang J.D., Song E. (2011). Microvesicles secreted by macrophages shuttle invasion-potentiating microRNAs into breast cancer cells. Mol. Cancer.

[116] Lan J., Sun L., Xu F., Liu L., Hu F., Song D., Hou Z., Wu W., Luo X., Wang J. (2019). M2 Macrophage-Derived Exosomes Promote Cell Migration and Invasion in Colon Cancer. Cancer Res..

[117] Erez N., Truitt M., Olson P., Arron S.T., Hanahan D. (2010). Cancer-Associated Fibroblasts Are Activated in Incipient Neoplasia to Orchestrate Tumor-Promoting Inflammation in an NF-kappaB-Dependent Manner. Cancer Cell.

[118] Josson S., Gururajan M., Sung S.Y., Hu P., Shao C., Zhau H.E., Liu C., Lichterman J., Duan P., Li Q. (2015). Stromal fibroblast-derived miR-409 promotes epithelial-to-mesenchymal transition and prostate tumorigenesis. Oncogene.

[119] Fong M.Y., Zhou W., Liu L., Alontaga A.Y., Chandra M., Ashby J., Chow A., O’Connor S.T., Li S., Chin A.R. (2015). Breast-cancer-secreted miR-122 reprograms glucose metabolism in premetastatic niche to promote metastasis. Nat. Cell Biol..

[120] Fang T., Lv H., Lv G., Li T., Wang C., Han Q., Yu L., Su B., Guo L., Huang S. (2018). Tumor-derived exosomal miR-1247-3p induces cancer-associated fibroblast activation to foster lung metastasis of liver cancer. Nat. Commun..

[121] Zhang L., Zhang S., Yao J., Lowery F.J., Zhang Q., Huang W.C., Li P., Li M., Wang X., Zhang C. (2015). Microenvironment-induced PTEN loss by exosomal microRNA primes brain metastasis outgrowth. Nature.

[122] Sakha S., Muramatsu T., Ueda K., Inazawa J. (2016). Exosomal microRNA miR-1246 induces cell motility and invasion through the regulation of DENND2D in oral squamous cell carcinoma. Sci. Rep..

[123] Nam J.W., Rissland O.S., Koppstein D., Abreu-Goodger C., Jan C.H., Agarwal V., Yildirim M.A., Rodriguez A., Bartel D.P. (2014). Global analyses of the effect of different cellular contexts on microRNA targeting. Mol. Cell.

[124] Clark P.M., Loher P., Quann K., Brody J., Londin E.R., Rigoutsos I. (2014). Argonaute CLIP-Seq reveals miRNA targetome diversity across tissue types. Sci. Rep..

[125] Ascano M., Hafner M., Cekan P., Gerstberger S., Tuschl T. (2012). Identification of RNA-protein interaction networks using PAR-CLIP. Wiley Interdiscip. Rev. RNA.

[126] Bracken C.P., Li X., Wright J.A., Lawrence D.M., Pillman K.A., Salmanidis M., Anderson M.A., Dredge B.K., Gregory P.A., Tsykin A. (2014). Genome-wide identification of miR-200 targets reveals a regulatory network controlling cell invasion. EMBO J..

[127] Hamilton M.P., Rajapakshe K.I., Bader D.A., Cerne J.Z., Smith E.A., Coarfa C., Hartig S.M., McGuire S.E. (2016). The Landscape of microRNA Targeting in Prostate Cancer Defined by AGO-PAR-CLIP. Neoplasia.

[128] Fletcher C.E., Sulpice E., Combe S., Shibakawa A., Leach D.A., Hamilton M.P., Chrysostomou S.L., Sharp A., Welti J., Yuan W. (2019). Androgen receptor-modulatory microRNAs provide insight into therapy resistance and therapeutic targets in advanced prostate cancer. Oncogene.

[129] He L., Thomson J.M., Hemann M.T., Hernando-Monge E., Mu D., Goodson S., Powers S., Cordon-Cardo C., Lowe S.W., Hannon G.J. (2005). A microRNA polycistron as a potential human oncogene. Nature.

[130] Akao Y., Nakagawa Y., Naoe T. (2006). let-7 microRNA functions as a potential growth suppressor in human colon cancer cells. Biol. Pharm. Bull..

[131] Bonci D., Coppola V., Musumeci M., Addario A., Giuffrida R., Memeo L., D’Urso L., Pagliuca A., Biffoni M., Labbaye C. (2008). The miR-15a-miR-16-1 cluster controls prostate cancer by targeting multiple oncogenic activities. Nat. Med..

[132] Garzon R., Liu S., Fabbri M., Liu Z., Heaphy C.E., Callegari E., Schwind S., Pang J., Yu J., Muthusamy N. (2009). MicroRNA-29b induces global DNA hypomethylation and tumor suppressor gene reexpression in acute myeloid leukemia by targeting directly DNMT3A and 3B and indirectly DNMT1. Blood.

[133] Trang P., Medina P.P., Wiggins J.F., Ruffino L., Kelnar K., Omotola M., Homer R., Brown D., Bader A.G., Weidhaas J.B. (2010). Regression of murine lung tumors by the let-7 microRNA. Oncogene.

[134] Klein U., Lia M., Crespo M., Siegel R., Shen Q., Mo T., Ambesi-Impiombato A., Califano A., Migliazza A., Bhagat G. (2010). The DLEU2/miR-15a/16-1 cluster controls B cell proliferation and its deletion leads to chronic lymphocytic leukemia. Cancer Cell.

[135] Zhao J.J., Lin J., Lwin T., Yang H., Guo J., Kong W., Dessureault S., Moscinski L.C., Rezania D., Dalton W.S. (2010). microRNA expression profile and identification of miR-29 as a prognostic marker and pathogenetic factor by targeting CDK6 in mantle cell lymphoma. Blood.

[136] Rupaimoole R., Slack F.J. (2017). MicroRNA therapeutics: Towards a new era for the management of cancer and other diseases. Nat. Rev. Drug Discov..

[137] Hanna J., Hossain G.S., Kocerha J. (2019). The Potential for microRNA Therapeutics and Clinical Research. Front. Genet..

[138] Liu Y.P., Vink M.A., Westerink J.T., Ramirez de Arellano E., Konstantinova P., Ter Brake O., Berkhout B. (2010). Titers of lentiviral vectors encoding shRNAs and miRNAs are reduced by different mechanisms that require distinct repair strategies. RNA.

[139] Lou W., Chen Q., Ma L., Liu J., Yang Z., Shen J., Cui Y., Bian X.W., Qian C. (2013). Oncolytic adenovirus co-expressing miRNA-34a and IL-24 induces superior antitumor activity in experimental tumor model. J. Mol. Med..

[140] Collins S.A., Guinn B.A., Harrison P.T., Scallan M.F., O’Sullivan G.C., Tangney M. (2008). Viral vectors in cancer immunotherapy: Which vector for which strategy?. Curr. Gene Ther..

[141] Campani V., Salzano G., Lusa S., De Rosa G. (2016). Lipid Nanovectors to Deliver RNA Oligonucleotides in Cancer. Nanomaterials.

[142] Ando H., Okamoto A., Yokota M., Asai T., Dewa T., Oku N. (2013). Polycation liposomes as a vector for potential intracellular delivery of microRNA. J. Gene Med..

[143] Dai Y., Zhang X. (2019). MicroRNA Delivery with Bioreducible Polyethylenimine as a Non-Viral Vector for Breast Cancer Gene Therapy. Macromol. Biosci..

[144] Li Y., Dai Y., Zhang X., Chen J. (2017). Three-layered polyplex as a microRNA targeted delivery system for breast cancer gene therapy. Nanotechnology.

[145] Pecot C.V., Rupaimoole R., Yang D., Akbani R., Ivan C., Lu C., Wu S., Han H.D., Shah M.Y., Rodriguez-Aguayo C. (2013). Tumour angiogenesis regulation by the miR-200 family. Nat. Commun..

[146] Jiang Q., Yuan Y., Gong Y., Luo X., Su X., Hu X., Zhu W. (2019). Therapeutic delivery of microRNA-143 by cationic lipoplexes for non-small cell lung cancer treatment in vivo. J. Cancer Res. Clin. Oncol..

[147] Chang H., Yi B., Ma R., Zhang X., Zhao H., Xi Y. (2016). CRISPR/cas9, a novel genomic tool to knock down microRNA in vitro and in vivo. Sci. Rep..

[148] Aquino-Jarquin G. (2017). Emerging Role of CRISPR/Cas9 Technology for MicroRNAs Editing in Cancer Research. Cancer Res..

[149] Yang J., Meng X., Pan J., Jiang N., Zhou C., Wu Z., Gong Z. (2018). CRISPR/Cas9-mediated noncoding RNA editing in human cancers. RNA Biol..

[150] Huo W., Zhao G., Yin J., Ouyang X., Wang Y., Yang C., Wang B., Dong P., Wang Z., Watari H. (2017). Lentiviral CRISPR/Cas9 vector mediated miR-21 gene editing inhibits the epithelial to mesenchymal transition in ovarian cancer cells. J. Cancer.

[151] El Fatimy R., Subramanian S., Uhlmann E.J., Krichevsky A.M. (2017). Genome Editing Reveals Glioblastoma Addiction to MicroRNA-10b. Mol. Ther. J. Am. Soc. Gene Ther..

[152] Esposito C.L., Cerchia L., Catuogno S., De Vita G., Dassie J.P., Santamaria G., Swiderski P., Condorelli G., Giangrande P.H., de Franciscis V. (2014). Multifunctional aptamer-miRNA conjugates for targeted cancer therapy. Mol. Ther. J. Am. Soc. Gene Ther..

[153] Cheng C.J., Bahal R., Babar I.A., Pincus Z., Barrera F., Liu C., Svoronos A., Braddock D.T., Glazer P.M., Engelman D.M. (2015). MicroRNA silencing for cancer therapy targeted to the tumor microenvironment. Nature.

[154] Almanza G., Rodvold J.J., Tsui B., Jepsen K., Carter H., Zanetti M. (2018). Extracellular vesicles produced in B cells deliver tumor suppressor miR-335 to breast cancer cells disrupting oncogenic programming in vitro and in vivo. Sci. Rep..

[155] Van Roosbroeck K., Fanini F., Setoyama T., Ivan C., Rodriguez-Aguayo C., Fuentes-Mattei E., Xiao L., Vannini I., Redis R.S., D’Abundo L. (2017). Combining Anti-Mir-155 with Chemotherapy for the Treatment of Lung Cancers. Clin. Cancer Res..

[156] Malhotra M., Sekar T.V., Ananta J.S., Devulapally R., Afjei R., Babikir H.A., Paulmurugan R., Massoud T.F. (2018). Targeted nanoparticle delivery of therapeutic antisense microRNAs presensitizes glioblastoma cells to lower effective doses of temozolomide in vitro and in a mouse model. Oncotarget.

[157] Devulapally R., Sekar N.M., Sekar T.V., Foygel K., Massoud T.F., Willmann J.K., Paulmurugan R. (2015). Polymer nanoparticles mediated codelivery of antimiR-10b and antimiR-21 for achieving triple negative breast cancer therapy. ACS Nano.

[158] Cortez M.A., Valdecanas D., Zhang X., Zhan Y., Bhardwaj V., Calin G.A., Komaki R., Giri D.K., Quini C.C., Wolfe T. (2014). Therapeutic delivery of miR-200c enhances radiosensitivity in lung cancer. Mol. Ther. J. Am. Soc. Gene Ther..

[159] Yin P.T., Pongkulapa T., Cho H.Y., Han J., Pasquale N.J., Rabie H., Kim J.H., Choi J.W., Lee K.B. (2018). Overcoming Chemoresistance in Cancer via Combined MicroRNA Therapeutics with Anticancer Drugs Using Multifunctional Magnetic Core-Shell Nanoparticles. ACS Appl. Mater. Interfaces.

[160] Rui M., Qu Y., Gao T., Ge Y., Feng C., Xu X. (2017). Simultaneous delivery of anti-miR21 with doxorubicin prodrug by mimetic lipoprotein nanoparticles for synergistic effect against drug resistance in cancer cells. Int. J. Nanomed..

[161] Wang F., Zhang L., Bai X., Cao X., Jiao X., Huang Y., Li Y., Qin Y., Wen Y. (2018). Stimuli-Responsive Nanocarrier for Co-delivery of MiR-31 and Doxorubicin To Suppress High MtEF4 Cancer. ACS Appl. Mater. Interfaces.

[162] Yao C., Liu J., Wu X., Tai Z., Gao Y., Zhu Q., Li J., Zhang L., Hu C., Gu F. (2016). Reducible self-assembling cationic polypeptide-based micelles mediate co-delivery of doxorubicin and microRNA-34a for androgen-independent prostate cancer therapy. J. Control. Release.

[163] Dai X., Fan W., Wang Y., Huang L., Jiang Y., Shi L., McKinley D., Tan W., Tan C. (2016). Combined Delivery of Let-7b MicroRNA and Paclitaxel via Biodegradable Nanoassemblies for the Treatment of KRAS Mutant Cancer. Mol. Pharm..

[164] Uz M., Kalaga M., Pothuraju R., Ju J., Junker W.M., Batra S.K., Mallapragada S., Rachagani S. (2019). Dual delivery nanoscale device for miR-345 and gemcitabine co-delivery to treat pancreatic cancer. J. Control. Release.

[165] Mondal G., Almawash S., Chaudhary A.K., Mahato R.I. (2017). EGFR-Targeted Cationic Polymeric Mixed Micelles for Codelivery of Gemcitabine and miR-205 for Treating Advanced Pancreatic Cancer. Mol. Pharm..

[166] Xu J., Sun J., Ho P.Y., Luo Z., Ma W., Zhao W., Rathod S.B., Fernandez C.A., Venkataramanan R., Xie W. (2019). Creatine based polymer for codelivery of bioengineered MicroRNA and chemodrugs against breast cancer lung metastasis. Biomaterials.

[167] Bader A.G. (2012). miR-34—A microRNA replacement therapy is headed to the clinic. Front. Genet..

[168] Reid G., Kao S.C., Pavlakis N., Brahmbhatt H., MacDiarmid J., Clarke S., Boyer M., van Zandwijk N. (2016). Clinical development of TargomiRs, a miRNA mimic-based treatment for patients with recurrent thoracic cancer. Epigenomics.

[169] Van Zandwijk N., Pavlakis N., Kao S.C., Linton A., Boyer M.J., Clarke S., Huynh Y., Chrzanowska A., Fulham M.J., Bailey D.L. (2017). Safety and activity of microRNA-loaded minicells in patients with recurrent malignant pleural mesothelioma: A first-in-man, phase 1, open-label, dose-escalation study. Lancet Oncol..

[170] Haghi M., Taha M.F., Javeri A. (2019). Suppressive effect of exogenous miR-16 and miR-34a on tumorigenesis of breast cancer cells. J. Cell. Biochem..

[171] Xu M., Li D., Yang C., Ji J.S. (2018). MicroRNA-34a Inhibition of the TLR Signaling Pathway Via CXCL10 Suppresses Breast Cancer Cell Invasion and Migration. Cell. Physiol. Biochem. Int. J. Exp. Cell. Physiol. Biochem. Pharmacol..

[172] Wang T.H., Yeh C.T., Ho J.Y., Ng K.F., Chen T.C. (2016). OncomiR miR-96 and miR-182 promote cell proliferation and invasion through targeting ephrinA5 in hepatocellular carcinoma. Mol. Carcinog..

[173] Han L., Chen W., Xia Y., Song Y., Zhao Z., Cheng H., Jiang T. (2018). MiR-101 inhibits the proliferation and metastasis of lung cancer by targeting zinc finger E-box binding homeobox 1. Am. J. Transl. Res..

[174] Ji H., Sang M., Liu F., Ai N., Geng C. (2019). miR-124 regulates EMT based on ZEB2 target to inhibit invasion and metastasis in triple-negative breast cancer. Pathol. Res. Pract..

[175] Zhao L., Li R., Xu S., Li Y., Zhao P., Dong W., Liu Z., Zhao Q., Tan B. (2018). Tumor suppressor miR-128-3p inhibits metastasis and epithelial-mesenchymal transition by targeting ZEB1 in esophageal squamous-cell cancer. Acta Biochim. Et Biophys. Sin..

[176] Liu B., Du R., Zhou L., Xu J., Chen S., Chen J., Yang X., Liu D.X., Shao Z.M., Zhang L. (2018). miR-200c/141 Regulates Breast Cancer Stem Cell Heterogeneity via Targeting HIPK1/beta-Catenin Axis. Theranostics.

[177] Li J., Xia L., Zhou Z., Zuo Z., Xu C., Song H., Cai J. (2018). MiR-186-5p upregulation inhibits proliferation, metastasis and epithelial-to-mesenchymal transition of colorectal cancer cell by targeting ZEB1. Arch. Biochem. Biophys..

[178] Sun G., Liu M., Han H. (2019). Overexpression of microRNA-190 inhibits migration, invasion, epithelial-mesenchymal transition, and angiogenesis through suppression of protein kinase B-extracellular signal-regulated kinase signaling pathway via binding to stanniocalicin 2 in breast cancer. J. Cell. Physiol..

[179] Yu Y., Luo W., Yang Z.J., Chi J.R., Li Y.R., Ding Y., Ge J., Wang X., Cao X.C. (2018). miR-190 suppresses breast cancer metastasis by regulation of TGF-beta-induced epithelial-mesenchymal transition. Mol. Cancer.

[180] Bian X., Liang Z., Feng A., Salgado E., Shim H. (2018). HDAC inhibitor suppresses proliferation and invasion of breast cancer cells through regulation of miR-200c targeting CRKL. Biochem. Pharmacol..

[181] Liu J., He D., Zhang G.J. (2018). Bioluminescence Imaging for Monitoring miR-200c Expression in Breast Cancer Cells and its Effects on Epithelial-Mesenchymal Transition Progress in Living Animals. Mol. Imaging Biol. Mib Off. Publ. Acad. Mol. Imaging.

[182] Seo S., Moon Y., Choi J., Yoon S., Jung K.H., Cheon J., Kim W., Kim D., Lee C.H., Kim S.W. (2019). The GTP binding activity of transglutaminase 2 promotes bone metastasis of breast cancer cells by downregulating microRNA-205. Am. J. Cancer Res..

[183] Wu L., Zhang Y., Huang Z., Gu H., Zhou K., Yin X., Xu J. (2019). MiR-409-3p Inhibits Cell Proliferation and Invasion of Osteosarcoma by Targeting Zinc-Finger E-Box-Binding Homeobox-1. Front. Pharmacol..

[184] Guo S.J., Zeng H.X., Huang P., Wang S., Xie C.H., Li S.J. (2018). MiR-508-3p inhibits cell invasion and epithelial-mesenchymal transition by targeting ZEB1 in triple-negative breast cancer. Eur. Rev. Med. Pharmacol. Sci..

[185] Yao R., Zheng H., Wu L., Cai P. (2018). miRNA-641 inhibits the proliferation, migration, and invasion and induces apoptosis of cervical cancer cells by directly targeting ZEB1. Onco Targets Ther..

[186] Lee J.W., Guan W., Han S., Hong D.K., Kim L.S., Kim H. (2018). MicroRNA-708-3p mediates metastasis and chemoresistance through inhibition of epithelial-to-mesenchymal transition in breast cancer. Cancer Sci..

